# Predicting Short-Term Outcome of COVID-19 Pneumonia Using Deep Learning-Based Automatic Detection Algorithm Analysis of Serial Chest Radiographs

**DOI:** 10.3390/bioengineering12101054

**Published:** 2025-09-29

**Authors:** Chae Young Lim, Yoon Ki Cha, Kyeongman Jeon, Subin Park, Kyunga Kim, Myung Jin Chung

**Affiliations:** 1 Department of Radiology, Samsung Medical Center, Sungkyunkwan University School of Medicine, 81 Irwon-ro Gangnam-gu, Seoul 06351, Republic of Korea; 2 Division of Pulmonary and Critical Care Medicine, Department of Medicine, Samsung Medical Center, Sungkyunkwan University School of Medicine, Seoul 06351, Republic of Korea; 3 Department of Digital Health, Samsung Advanced Institute for Health Sciences and Technology, Sungkyunkwan University, Seoul 06351, Republic of Korea; 4 Department of Data Convergence & Future Medicine, Sungkyunkwan University School of Medicine, Seoul 06351, Republic of Korea; 5 Medical AI Research Center, Samsung Medical Center, Seoul 06351, Republic of Korea

**Keywords:** COVID-19, chest radiography, commercial AI, Grad-CAM, time-dependent receiver operating characteristic curve

## Abstract

This study aimed to evaluate short-term clinical outcomes in COVID-19 pneumonia patients using parameters derived from a commercial deep learning-based automatic detection algorithm (DLAD) applied to serial chest radiographs (CXRs). We analyzed 391 patients with COVID-19 who underwent serial CXRs during isolation at a residential treatment center (median interval: 3.57 days; range: 1.73–5.56 days). Patients were categorized into two groups: the improved group (*n* = 309), who completed the standard 7-day quarantine, and the deteriorated group (*n* = 82), who showed worsening symptoms, vital signs, or CXR findings. Using DLAD’s consolidation probability scores and gradient-weighted class activation mapping (Grad-CAM)-based localization maps, we quantified the consolidation area through heatmap segmentation. The weighted area was calculated as the sum of the consolidation regions’ areas, with each area weighted by its corresponding probability score. Change rates (Δ) were defined as per-day differences between consecutive measurements. Prediction models were developed using Cox proportional hazards regression and evaluated daily from day 1 to day 7 after the subsequent CXR acquisition. Among the imaging factors, baseline probability and ΔProbability, ΔArea, and ΔWeighted area were identified as prognostic indicators. The multivariate Cox model incorporating baseline probability and ΔWeighted area demonstrated optimal performance (C-index: 0.75, 95% Confidence Interval: 0.68–0.81; integrated calibration index: 0.03), with time-dependent AUROC (Area Under Receiver Operating Curve) values ranging from 0.74 to 0.78 across daily predictions. These findings suggest that the Δparameters of DLAD can aid in predicting short-term clinical outcomes in patients with COVID-19.

## 1. Introduction

The Coronavirus disease 2019 (COVID-19) international consensus statements proposed that radiologic examinations, such as chest radiography (CXR), can serve as an initial triage tool in healthcare settings facing resource constraints [1,2,3,4]. Following that, CXR has been frequently employed in assessing COVID-19 pneumonia and served as a cornerstone for monitoring the disease’s progression [2].

During the COVID-19 pandemic, South Korea established residential treatment centers (RTCs) to isolate and treat patients with mild COVID-19 symptoms [5]. These centers monitored patients and determined when hospital transfer was needed. To aid in interpreting patient chest X-rays (CXRs), many RTCs adopted commercial deep learning-based automatic detection algorithms (DLADs) [6], which had shown radiologist-level performance in detecting pneumonia, including COVID-19 cases [7,8,9].

While commercial AI systems have demonstrated strong performance in detecting thoracic abnormalities in images [10,11,12], their application has primarily focused on diagnostic classification rather than prognosis. Several studies have explored deep learning for COVID-19 prognostication, but most rely on proprietary in-house algorithms with limited accessibility. Notably, Shin et al. demonstrated the prognostic value of abnormality scores from a commercial AI system (Lunit INSIGHT CXR) in patients with COVID-19 [13]. However, that analysis was limited to single timepoint assessments, overlooking the dynamic changes in COVID-19 pneumonia that are visible in sequential images and known to indicate disease progression [14,15].

Gradient-weighted Class Activation Mapping (Grad-CAM) visualizes regions influencing deep neural network predictions [16]. In medical imaging, these heatmaps correspond with disease-affected areas [17,18], yet their use for tracking disease progression remains unexplored in COVID-19. We hypothesized that parameters from a commercial AI system, including both class probability scores and areas derived from Grad-CAM-based localization maps from sequential chest X-rays (CXRs), can better predict the outcome of patients with COVID-19 in RTCs. We quantitatively analyzed these parameters over time to evaluate the prognostic utility of commercial AI in COVID-19.

## 2. Materials and Methods

### 2.1. Patients, Data Collection, and Outcome Definition

**Figure 1:**
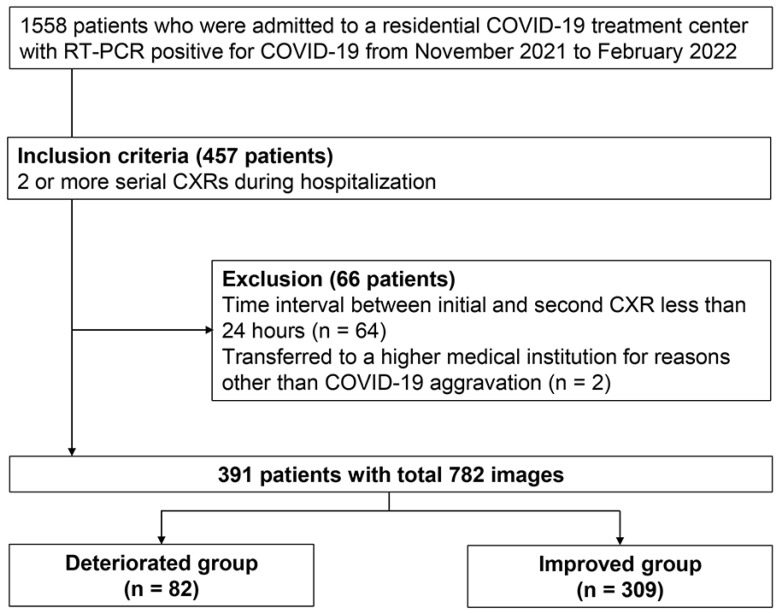
Study flowchart: Patient selection process for inclusion.

A total of 1558 patients were admitted to a RTC with a positive reverse transcription-polymerase chain reaction (RT-PCR) for COVID-19 between November 2021 and February 2022, a period when the COVID-19 delta variant had widely spread in South Korea [19]. At initial RTC screening, patients with findings of COVID-19 pneumonia (consolidations or ground-glass opacities) on CXR were directly referred to hospitals without RTC admission. Only asymptomatic patients who passed screening were admitted to RTC. Follow-up CXRs were performed when patients reported worsening symptoms, when they exhibited decreased oxygen saturation, or when initial findings required monitoring. Among RTC-admitted patients, 457 underwent at least two serial CXRs during their stay. We excluded 64 patients whose time intervals between initial and subsequent CXRs were less than a day to avoid extrapolation errors when scaling changes per day. Additionally, two patients transferred for non-COVID-19 reasons were excluded. This resulted in a final analysis cohort of 391 consecutive patients. A total of 910 CXR scans were obtained during their stay, of which 782 scans were used for analysis (Figure 1). We collected baseline clinical characteristics (age, sex, and comorbidities) and follow-up data (isolation period, serial CXRs, and reasons for leaving the RTC). The primary outcome was clinical deterioration after the subsequent CXR acquisition during the isolation, defined as patient transfer to higher medical institutions. Transfer criteria included: (1) presence of consolidation on follow-up CXR, (2) worsening respiratory symptoms such as dyspnea, or (3) vital sign instability including oxygen saturation below 94% on room air. While radiological findings were among the transfer criteria, we measured the outcome after the predictor assessment to maintain temporal separation. Improvement was defined as completing the standard 7-day quarantine period at the residential treatment center without clinical deterioration.

### 2.2. DLAD System for CXR Interpretation

The DLAD system (Lunit INSIGHT for Chest Radiography, version 3.1.4.1; Lunit, Seoul, Republic of Korea [https://insight.lunit.io], accessed on 29 September 2025) analyzes chest radiographs through lesion-specific output channels for 10 chest abnormalities (pneumothorax, mediastinal widening, pneumoperitoneum, nodule, consolidation, pleural effusion, atelectasis, fibrosis, calcification and cardiomegaly). These abnormalities were defined in accordance with the Fleischer Society glossary [20]. The system outputs: (1) class probability scores indicating the likelihood of each abnormality, and (2) Grad-CAM-based localization maps highlighting the corresponding detected regions [16,21].

### 2.3. Quantitative Image Analysis and Parameter Measurements

**Figure 2:**
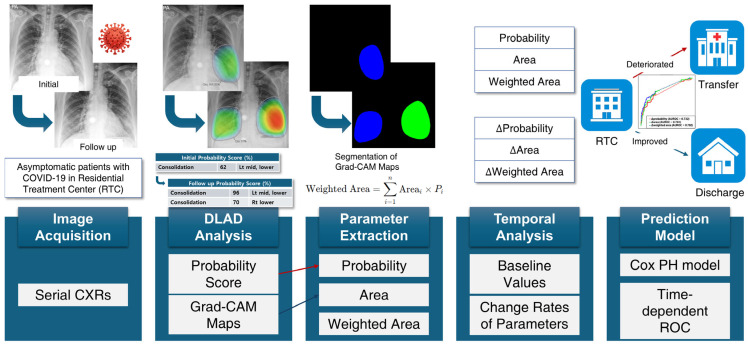
Quantitative image analysis pipeline. DLAD, deep learning automatic detection; CXR, chest X-ray; ROC, receiver operating characteristic; Grad-CAM, gradient-weighted class activation mapping; PH, proportional hazards.

**Figure 3:**
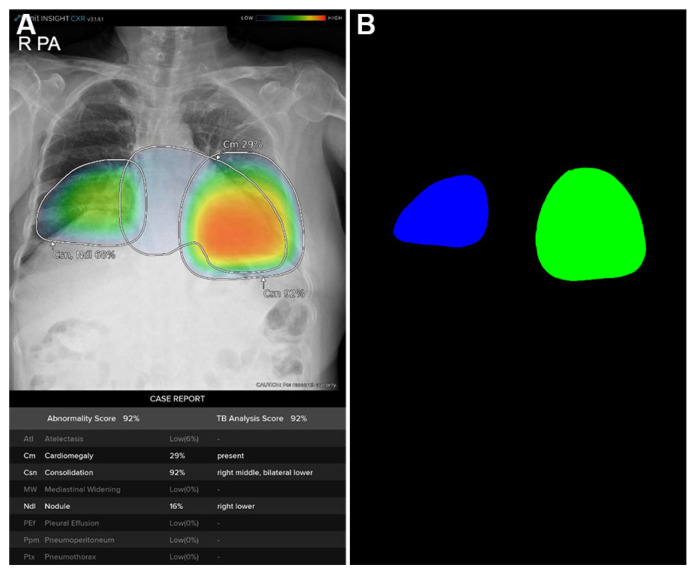
Quantitative areas of consolidation automatically calculated from the deep learning-based automatic detection algorithm (DLAD) localization map using OpenCV python program. (**A**) Localization map from the chest X-ray of a 77-year-old COVID-19 patient showing bilateral consolidation with annotations and abnormality scores/probabilities suggested by DLAD. (**B**) OpenCV program automatically detects and calculates quantitative area of each consolidation heatmap.

The quantitative image analysis pipeline is illustrated in Figure 2. The DLAD system analyzed CXRs with a median interval of 3.57 days (range: 1.73–5.56) between serial examinations. Using consolidation probability scores and Grad-CAM-based localization maps (Figure 3A), regions of interest (ROIs) were quantified through heatmap area segmentation using OpenCV Python program version 4.2.0 (Figure 3B). Segmentation quality was visually confirmed by a board-certified radiologist (BLINDED). The ‘area’ represented the fractional percentage of total ROI sizes relative to the entire CXR. We defined ‘probability’ as the maximum consolidation probability score among all ROIs. The ‘weighted area’ was calculated using the following equation:

$$\mathrm{Weighted}\;\mathrm{Area}=\sum_{i=1}^{n}\left({\mathrm{Area}}_{i}\times {Probability}_{i}\right)$$

where ${\mathrm{Area}}_{i}\;$ is the size of the *i*-th ROI and ${Probability}_{i}$ is its corresponding probability for consolidation.

These parameters were measured from both initial and the first subsequent CXRs. We measured change rates (Δ) for each parameter between initial and the first subsequent CXRs, scaled to a per-day basis for standardized comparison across patients with different intervals between imaging studies. Specifically,

$$\Delta \mathrm{Parameter}=\frac{{\mathrm{Parameter}}_{\mathrm{subsequent}}-{\mathrm{Parameter}}_{\mathrm{initial}}}{\mathrm{Days}\;\mathrm{between}\;\mathrm{CXRs}}$$

where parameter represents probability, area, or weighted area of consolidation. Both baseline and Δparameters were evaluated as potential predictors for clinical deterioration.

### 2.4. Statistical Analysis and Predictive Modeling

Clinical and imaging features were summarized as median with range for continuous variables or frequency with percentage for categorical variables. Between-group comparisons were performed using Student’s *t*-test when normality assumptions were met, or Mann–Whitney U test otherwise. Categorical variables were compared using chi-square tests under the assumption of adequate expected counts.

To assess generalized hazard over time, Cox proportional hazards regression was used to estimate hazard ratios (HRs) with 95% confidence intervals (CIs). The proportional hazards assumption was evaluated using Schoenfeld residuals with graphical inspection of β(t), which showed no major violations. Candidate predictors significant in univariable analysis were entered into multivariable Cox regression models, which were internally validated with 1000 bootstrap samples. Model discrimination was assessed using Harrell’s C-index. Further performance comparisons were conducted with calibration plots, the integrated calibration index (ICI), the median (E50) and 90th percentile (E90) of absolute differences between observed and predicted probabilities, and net benefit curves.

For time-varying performance assessment, time-dependent receiver operating characteristic (ROC) curve and area under the ROC curve (AUROC) analyses were performed using the cumulative incidence/dynamic controls (C/D) definition. Incident cases/dynamic controls (I/D) were also evaluated. Kaplan–Meier survival analyses and log-rank tests were additionally conducted for risk stratification, with cutoffs for ΔProbability, ΔArea, and ΔWeighted area determined by Youden’s indices from the time-dependent ROC curve.

All statistical analyses were performed using R software (version 4.1.3; R Foundation for Statistical Computing, Vienna, Austria) with the survival, timeROC, and rms packages.

## 3. Results

### 3.1. Patient Characteristics

A total of 391 patients with COVID-19 were included; 82 (21.0%) experienced clinical deterioration, with 60 (73.2%) of these showing consolidation on CXR. Table 1 summarizes characteristics of both improved (*n* = 309) and deteriorated (*n* = 82) groups. The median age was significantly higher in the deteriorated group (61 years vs. 49 years, *p* < 0.001). The prevalence of diabetes was significantly higher in the deteriorated group (18.29% vs. 9.39%, *p* = 0.04). The isolation period and interval between serial CXRs were significantly shorter in the deteriorated group (median 3.34 days vs. 7 days and 1.73 days vs. 3.74 days, respectively; all *p* < 0.001). Both baseline and Δimaging parameters were higher in the deteriorated group (*p* < 0.001 to *p* < 0.01).

### 3.2. Candidate Predictor Selection

In the univariable Cox-proportional hazard analysis, baseline probability, ΔProbability, ΔArea, and ΔWeighted area exhibited significant associations with clinical deterioration (HRs and 95% CIs: 1.01 [1.00–1.02], 1.05 [1.04–1.06], 1.33 [1.24–1.43], and 1.46 [1.35–1.58], respectively; see Table 2). ΔProbability, ΔArea, and ΔWeighted area demonstrated fair overall prognostic values (C-indices: 0.71, 0.73, and 0.75, respectively). These parameters also exhibited good performance in daily prediction (time-dependent AUROC from day 1 to day 7: 0.70–0.75, 0.71–0.76, and 0.74–0.78, respectively), while baseline probability, area, and weighted area did not discriminate (Figure S1). In the correlation analysis, ΔWeighted area exhibited strong positive correlations with ΔProbability and ΔArea (R = 0.87 and R = 0.85, respectively; *p* < 0.001 for both). Correlations between other parameters are provided in Table S1.

### 3.3. Predictive Modeling and Validation

Using the selected candidate predictors, we constructed three predictive models using multivariable Cox regressions. Firstly, ΔWeighted area was retained after employing a stepwise selection procedure (Model 1). Secondly, due to the high correlations between ΔWeighted area and the other two change parameters, they were placed in separate models as follows: Model 2 included baseline probability and ΔWeighted area; Model 3 included baseline probability, ΔProbability, and ΔArea. Other models incorporating paired baseline and Δparameters are provided in Table S2.

In the bootstrap-based internal validation, we evaluated and compared the performances of the three models. While the overall prognostic value of Model 1 appeared higher (C-indices and 95% CIs for Models 1, 2, and 3: 0.75 [0.69, 0.81], 0.745 [0.68, 0.81], and 0.729 [0.66, 0.80], respectively), the pair-wise differences in prognostic values were not significant (ΔC-indices and 95% CIs for Model 1 vs. Model 2, Model 1 vs. Model 3, and Model 2 vs. Model 3: 0.01 [−0.03, 0.03], 0.02 [−0.01, 0.05], and 0.02 [−0.01, 0.05], respectively). Models incorporating clinical predictors (age and diabetes) were also evaluated (Table S3). The C-indices of these models were not significantly different from the imaging-only model.

**Figure 4:**
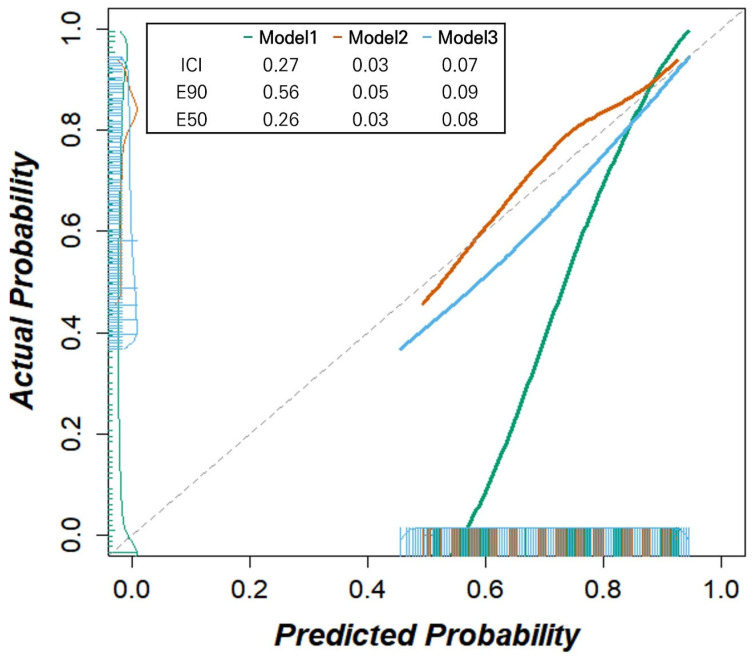
Calibration plots with integrated calibration indices (ICIs) of three deep learning-based automatic detection algorithm (DLAD) based models predicting clinical deterioration on day 2 in patients with COVID-19. Model 2 demonstrates the best calibration with the lowest ICI (0.03), E50 (0.03), and E90 (0.05) values, compared to Model 1 (ICI: 0.27, E50: 0.26, E90: 0.56) and Model 3 (ICI: 0.07, E50: 0.08, E90: 0.09). Model 1 shows significant deviation from the diagonal line, indicating poor calibration, while Model 3 shows slight overestimation of deterioration probability. Model 2 follows the diagonal line most closely, indicating good agreement between predicted and observed probabilities. Note: The diagonal dashed line represents perfect calibration. Integrated calibration index (ICI) represents calibration error metrics, where lower values indicate better calibration performance. E50 and E90 denote the median and 90th percentile of absolute differences between observed and predicted probabilities, respectively. Bars on the x-axis indicate the predicted probabilities of deterioration, while those on the y-axis denote the observed outcome frequencies within each bin.

**Figure 5:**
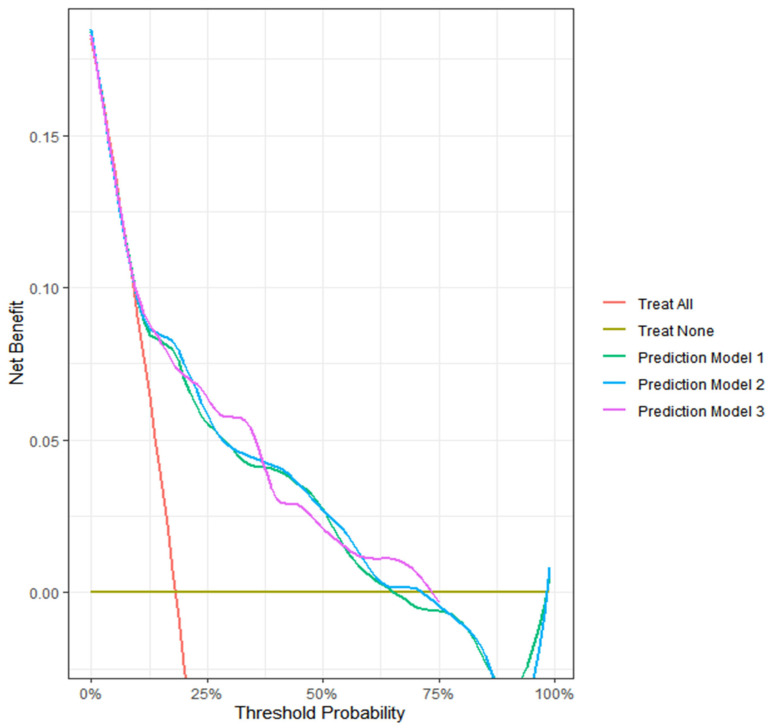
Decision curve analysis of three deep learning-based automatic detection algorithm (DLAD) based models predicting clinical deterioration on day 2 in patients with COVID-19.

We further assessed the reliability of our models using calibration plots and calibration measures for day 2 (Figure 4). While Model 1 exhibited significant deviation from the reference line and Model 3 showed an overestimation of the deterioration probability, Model 2 demonstrated the best calibration (ICIs for Model 1, 2, and 3: 0.27, 0.03, and 0.07, respectively, with lower values indicating better calibration). Similarly, Model 2 showed the lowest calibration errors (E50 for Models 1, 2, and 3: 0.26, 0.03, and 0.08; E90: 0.56, 0.05, and 0.09, respectively). Intervention strategies based on all three models exhibited larger net benefits than the ‘treat all’ and ‘treat none’ scenarios within the threshold probability range of 12.5% and 62.5% (Figure 5). The decision curve analysis highlights the clinical utility of our models.

**Figure 6:**
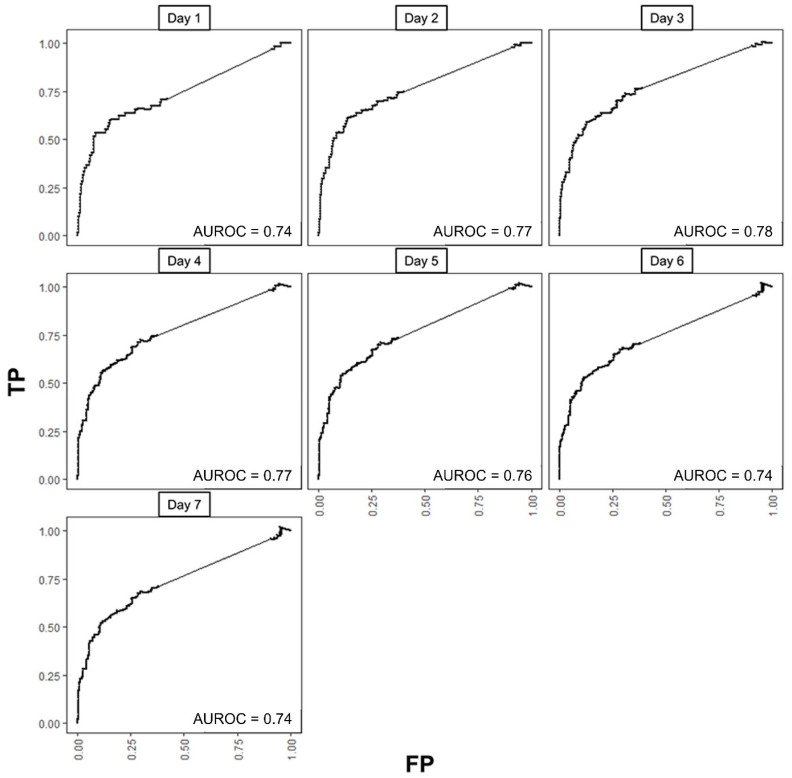
Time-dependent receiver operating characteristic curves (ROCs) for final multivariable Cox proportional hazards model (Model 2) with baseline probability and ΔWeighted area each day in the first week of isolation. TP, true positive rate; FP, false positive rate; AUROC area under ROC curve.

We selected Model 2 as our final predictive model because it demonstrated fairly good discrimination and clinical utility, along with better calibration performance compared to the other models. We investigated the time-specific predictivity of our final model using time-dependent ROC curves for each day in the first week of isolation (Figure 6) under the C/D definition. AUROCs ranged from 0.74 to 0.78, with values exceeding 0.76 from Day 2 to Day 5. Using Day 2 as the reference, no notable differences were observed across the other days during the week (all *p* > 0.05). Under the I/D definition, AUROCs were 0.74 (0.73–0.76) at Day 1, 0.93 (0.90–0.95) at Day 2, and 0.88 (0.82–0.89) at Day 3, then tapered thereafter (0.82 at Day 4; 0.72 at Day 5). I/D AUROC was not estimable on Days 6–7 due to the absence of incident events among those still at risk. Kaplan–Meier analyses stratified by the Day 2 C/D ROC cutoffs for ΔProbability, ΔArea, and ΔWeighted area demonstrated clear separation between high- and low-risk groups (Figure S2, all *p* < 0.001).

## 4. Discussion

The study identified several key variables derived from commercial DLAD of CXR —namely ΔProbability, ΔArea, and ΔWeighted area—for predicting deterioration or improvement of COVID-19 pneumonia in serial radiographs. Based on these predictors, multivariable Cox models were constructed, suggesting their potential to aid in prognosticating patients with COVID-19 referred to RTC. Calibration plots and decision curve analysis on day 2 (48 h) demonstrated the model’s potential clinical utility for early decision-making.

When considering both C/D and I/D definitions of time-dependent ROC, noteworthy implications emerge. I/D values remained high even after the early transfer window. This suggests that the DLAD-derived Δparameters captured prognostic signals beyond immediate imaging-related decisions of transfer, supporting the robustness of our model in time-varying prediction.

NIH COVID-19 treatment guidelines emphasize that antiviral therapy should be initiated as early as possible [22]. We adopted the 48 h prediction horizon because it represents the earliest stable time point at which outcomes can be assessed independently of the imaging input. Moreover, prior studies have also adopted 48 h benchmarks as clinically meaningful endpoints in COVID-19, [23,24], implying it as an evidence-based window for early intervention in patients at risk of deterioration.

Longitudinal assessment of COVID-19 pneumonia using deep learning with CXR has also been reported. Gourdeau et al. applied a deep learning framework to sequential chest radiographs and achieved an AUC of 0.81 for distinguishing worsening from improvement [25]. Similarly, Duanmu et al. demonstrated that deep learning analysis of longitudinal CXR data over 5 consecutive days predicted mortality with 85% accuracy and an AUC of 0.87 [15]. On the other side, CT-based prognostic studies have primarily focused on hospitalized patients with more severe disease, often using outcomes such as high-flow oxygen requirement, mechanical ventilation, or death [26], or correlating CT severity scores with inflammatory markers and mortality [27,28,29]. Mruk et al. further validated various semi-quantitative CT severity scoring systems, supporting their prognostic value in clinical practice [30].

Recent advances in explainable AI further contextualize our findings. Grad-CAM has been successfully applied in diverse chest imaging tasks, including improved detection of pulmonary diseases [31,32] and in other medical imaging modalities, highlighting the maturity of saliency-based interpretation tools and explainable AI. Beyond radiology, explainable machine learning approaches have demonstrated value in other real-world domains [33]. These advances in AI explainability support clinical applicability and real-world relevance of our DLAD-based framework.

## 5. Future Work and Limitations

This study has several limitations. First, the RTC setting provided only limited clinical data (symptoms, vital signs, SpO_2_, comorbidities, and CXRs). While this setting highlights the utility of DLAD in helping rapid interpretation of CXRs, it also constrains our study by preventing the integration of more comprehensive laboratory data. Second, our cohort came from a single RTC during the Delta variant phase, limiting generalizability. Including multicenter RTC data, especially those covering the Omicron period, would improve applicability to newer populations and variants. External validation across them will be essential to establish the reliability and clinical utility of our findings. Third, follow-up CXRs were obtained with non-standardized schedules and patients with <1 day intervals were excluded, introducing potential selection bias.

Future work should focus on extending the clinical utility of our approach. Our quantitative analysis can be streamlined into an automated pipeline that directly integrates DLAD outputs such as Grad-CAM maps and abnormality probabilities. Embedding this into commercial AI platforms would enable automated generation of longitudinal risk scores. Since our decision curve analyses suggest potential for optimizing referral and discharge decisions, this information could provide critical clinical decision support. Future studies, including evaluation of cost-effectiveness thresholding, should further assess its value for real-world implementation [34].

## 6. Conclusions

We developed and evaluated prediction models using Δparameters from a commercial DLAD to forecast short-term outcomes in quarantined patients with COVID-19 at RTCs. Our results suggest that commercial DLADs can assist in clinical decision-making through their prognostic capability, particularly in resource-limited RTC settings. Further investigation is required to fully assess their clinical impact.

## Figures and Tables

**Table 1:** Patient characteristics.

Variable	Total (*N* = 391)	Improved Group (*N* = 309)	Deteriorated Group (*N* = 82)	*p*-Value
*Clinical features*				
Age (years)	52 (31, 66.5)	49 (28, 65)	61 (46, 70)	<0.001
Sex				0.99
Male	213 (54.48%)	168 (54.37%)	45 (54.88%)	
Female	178 (45.52%)	141 (45.63%)	37 (45.12%)	
Hypertension	88 (22.51%)	66 (21.36%)	22 (26.83%)	0.37
Diabetes	44 (11.25%)	29 (9.39%)	15 (18.29%)	0.04
Cardiovascular Disease	10 (2.56%)	7 (2.27%)	3 (3.66%)	0.44
Isolation period (day)	7 (7, 7)	7 (7, 7)	3.34 (1.71, 4.69)	<0.001
Serial CXR interval (day)	3.57 (1.73, 5.56)	3.74 (1.86, 5.7)	1.73 (1.58, 3.43)	<0.001
*Imaging features*				
* Baseline, initial*				
Probability	0 (0, 33.5)	0 (0, 23)	0 (0, 52.5)	<0.01
Area	0 (0, 3.85)	0 (0, 2.86)	1.56 (0, 5.94)	<0.001
Weighted area	0 (0, 1.12)	0 (0, 0.54)	0 (0, 3.09)	<0.01
* Change rate*				
ΔProbability	0 (0, 4.63)	0 (0, 1.59)	7.63 (0, 20.26)	<0.001
ΔArea	0 (0, 0.86)	0 (0, 0.36)	1.16 (0, 3.54)	<0.001
ΔWeighted area	0 (0, 0.38)	0 (0, 0.06)	1.08 (0, 3.4)	<0.001

Categorical variables were summarized by the number of patients with percentage in parentheses. Numeric variables were summarized by median with range in parentheses. ΔProbability, ΔArea, ΔWeighted area represent the change rates per day from initial to subsequent CXRs in probability, area and weighted area of consolidation, respectively.

**Table 2:** Univariable and multivariable Cox proportional hazard regression analyses predicting the deteriorated group.

Variables	Univariable Analysis				Model 1			Model 2			Model 3		
	HR	95% CI	*p*-Value	C-Index	HR	95% CI	*p*-Value	HR	95% CI	*p*-Value	HR	95% CI	*p*-Value
Probability, baseline	1.01	1.00–1.02	0.01	0.58				1	1.00–1.01	0.209	1.02	1.01–1.02	<0.001
Area, baseline	1.03	1.00–1.07	0.08	0.59									
Weighted area, baseline	1.03	0.99–1.07	0.90	0.57									
ΔProbability	1.05	1.04–1.06	<0.001	0.71				-	-	-	1.05	1.03–1.08	<0.001
ΔArea	1.33	1.24–1.43	<0.001	0.73				-	-	-	1.05	0.93–1.20	0.43
ΔWeighted area	1.46	1.35–1.58	<0.001	0.75	1.46	1.35–1.58	<0.001	1.44	1.32–1.56	<0.001	-	-	-
C-index in model development					0.75 (0.69, 0.81)			0.74 (0.68, 0.81)			0.72 (0.65, 0.79)		
C-index in internal validation					0.75 (0.69, 0.81)			0.74 (0.68, 0.81)			0.73 (0.66, 0.79)		

HR, hazards ratio; CI confidence interval. ΔProbability, ΔArea, ΔWeighted area represent the change rates per day from initial to subsequent CXRs in Probability, Area and Weighted area, respectively. CXRs in Probability, Area and Weighted area of consolidation, respectively.

## Data Availability

Due to the sensitive nature of the data and privacy concerns, the data supporting the findings of this study are not publicly available. Details about the data and how they can be accessed are available from the corresponding author upon reasonable request.

## Supplementary Materials

The following supporting information can be downloaded at: https://www.mdpi.com/article/10.3390/bioengineering12101054/s1, Figure S1: Time-dependent receiver operating characteristic curves (ROCs) for the change rates of three imaging parameters each day in the first week of hospitalization; Figure S2: Kaplan–Meier curves for all patients with COVID-19 (event: transfer to higher medical institution), (A), and risk group with cutoff 0.767 for Δprobability (B), with cutoff 0.220 for Δarea (C), and with cutoff 0.668 for Δarea*probability (D); Table S1: Correlation matrix of imaging parameters derived from deep-learning-based automatic detection system; Table S2: Cox proportional hazard models predicting the deteriorated group with paired baseline and change rates of each parameter; Table S3: Cox proportional hazard models predicting the deteriorated group with incorporated clinical and imaging variables.
