# Supplementary material for: Predicting Short-Term Outcome of COVID-19 Pneumonia Using Deep Learning-Based Automatic Detection Algorithm Analysis of Serial Chest Radiographs
Source: Bioengineering (Basel). 2025 Sep 29;12(10):1054. doi: 10.3390/bioengineering12101054 (PMC12561940; doi:10.3390/bioengineering12101054)
Supplement: Supplementary file 1 [file bioengineering-12-01054-s001.zip › bioengineering-3861581-supplementary.pdf]

Supplementary Table S1. Correlation matrix of imaging parameters derived from deep-learning-based automatic detection system.

	**Δprobability**	**Δarea**	**Δweighted area**	**Probability**	**Area**	**Weighted area**
Δprobability	1					
Δarea	0.73	1				
Δweighted area	0.87	0.85	1			
Probability	-0.17	0.03	-0.04	1		
Area	-0.11	-0.05	-0.01	0.88	1	
Weighted area	-0.18	0.02	-0.05	0.99	0.88	1

ΔProbability, ΔArea, and ΔWeighted area represent the change rates per day from initial to subsequent CXRs in Probability, Area, and Weighted area, respectively.

Supplementary Table S2. Cox proportional hazard models predicting the deteriorated group with paired baseline and change rates of each parameter.

Variables	Univariable Analysis				Model 1			Model 2			Model 3		
	HR	95% CI	*P*-value	C-index	HR	95% CI	*P*-value	HR	95% CI	*P*-value	HR	95% CI	*P*-value
Probability, baseline	1.01	1.00-1.02	0.007	0.579	1.02	1.01–1.02	<0.001						
Area, baseline	1.03	1.00-1.07	0.079	0.592				1.04	1.00–1.08	0.038			
Weighted area, baseline	1.03	0.99-1.07	0.900	0.569							1.03	0.97–1.08	0.353
ΔProbability	1.05	1.04-1.06	<0.001	0.706	1.06	1.05–1.07	<0.001						
ΔArea	1.33	1.24-1.43	<0.001	0.731				1.33	1.24–1.43	<0.001			
ΔWeighted area	1.46	1.35-1.58	<0.001	0.751							1.45	1.34–1.57	<0.001
C-index in model development					0.716 (0.646–0.787)			0.737 (0.674–0.800)			0.750 (0.687–0.812)		
C-index in internal validation					0.725 (0.658–0.792)			0.738 (0.674–0.798)			0.744 (0.673–0.808)		

Comparison of three Cox proportional hazard models: Model 1 (baseline probability + Δprobability), Model 2 (baseline area + Δarea), and Model 3 (baseline weighted area + Δweighted area).

HR = hazards ratio, CI = confidence interval.

Δ represents the change rate per day between initial and subsequent CXRs for each parameter.

C-indices are presented with 95% confidence intervals in parentheses.

Supplementary Table S3. Cox proportional hazard models predicting the deteriorated group with incorporated clinical and imaging variables.

Variables	Univariable Analysis				Model 1 (reference)			Model 2			Model 3		
	**HR**	**95% CI**	***P*-value**	**C-index**	**HR**	**95% CI**	***P*-value**	**HR**	**95% CI**	***P*-value**	**HR**	**95% CI**	***P*-value**
Probability, baseline	1.01	1.00-1.02	0.007	0.579				1.00	0.99–1.01	0.598			
ΔWeighted area	1.46	1.35-1.58	<0.001	0.751	1.46	1.35-1.58	<0.001	1.41	1.30–1.54	<0.001	1.42	1.31–1.54	<0.001
Age	1.02	1.01–1.03	0.002	0.599				1.01	1.00–1.03	0.094	1.01	1.00–1.03	0.065
Diabetes	1.87	1.06–3.31	0.028	0.540				1.21	0.66–2.20	0.543	1.25	0.69–2.24	0.465
C-index in model development					0.751 (0.690–0.812)			0.769 (0.708–0.830)			0.765 (0.704–0.826)		
C-index in internal validation					0.752 (0.692–0.813)			0.769 (0.708–0.828)			0.766 (0.706–0.823)		

Model 1 (baseline probability + Δweighted area), Model 2 (baseline probability + Δweighted area + age + diabetes), and Model 3 (baseline probability + Δweighted area + Δarea + age + diabetes).

HR = hazards ratio, CI = confidence interval.

Δ represents the change rate per day between initial and subsequent CXRs for each parameter.

C-indices are presented with 95% confidence intervals in parentheses.

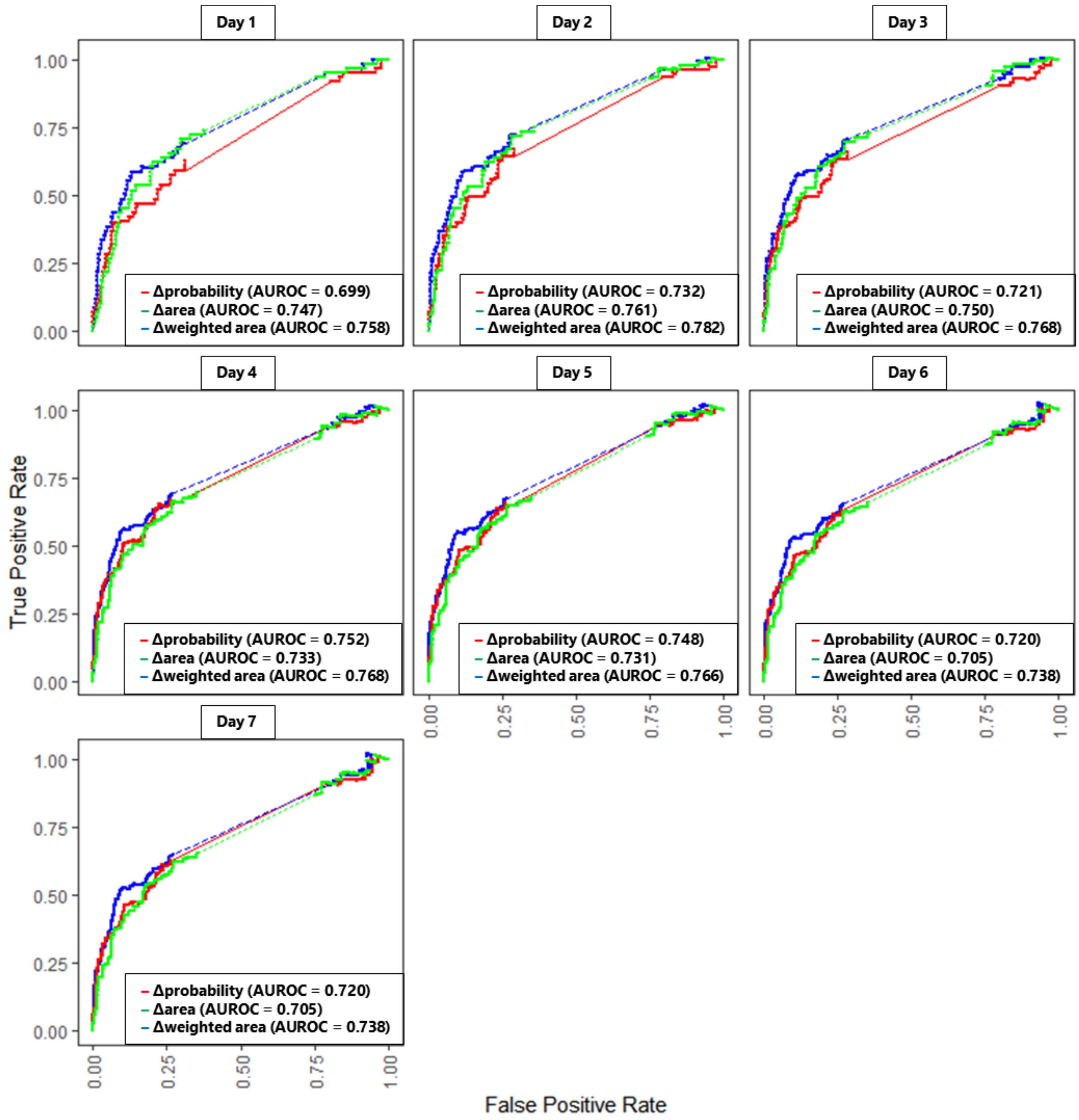

Supplementary Figure S1. Time-dependent receiver operating characteristic curves (ROCs) for the change rates of three imaging parameters each day in the first week of hospitalization. AUROC = area under ROC curve. ΔProbability, ΔArea, and ΔWeighted area represent the

change rates per day from initial to subsequent CXRs in Probability, Area, and Weighted area, respectively.

Supplementary Figure S2. Kaplan–Meier curves for all patients with COVID-19 (event: transfer to higher medical institution), (A), and risk group with cutoff 0.767 for Δprobability (B), with cutoff 0.220 for Δarea (C), and with cutoff 0.668 for Δarea*probability (D).

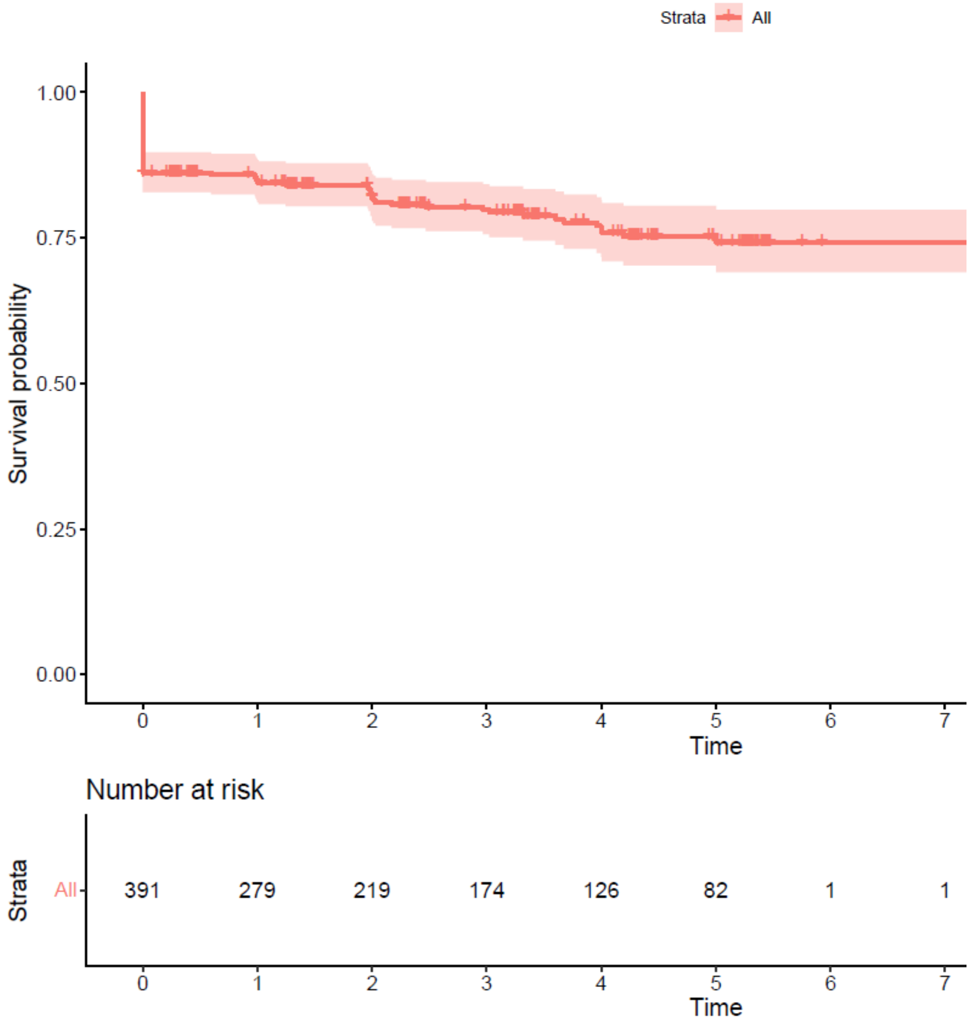

A. All participants.

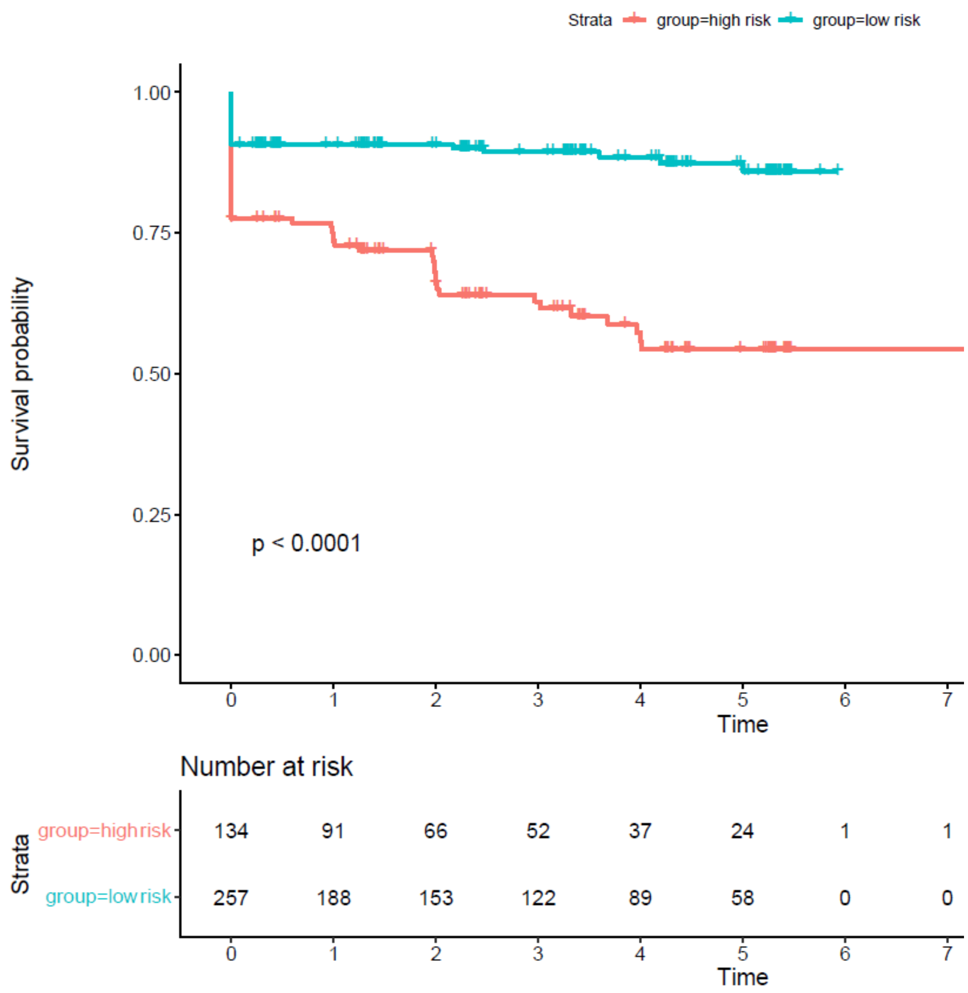

B. Δ probability.

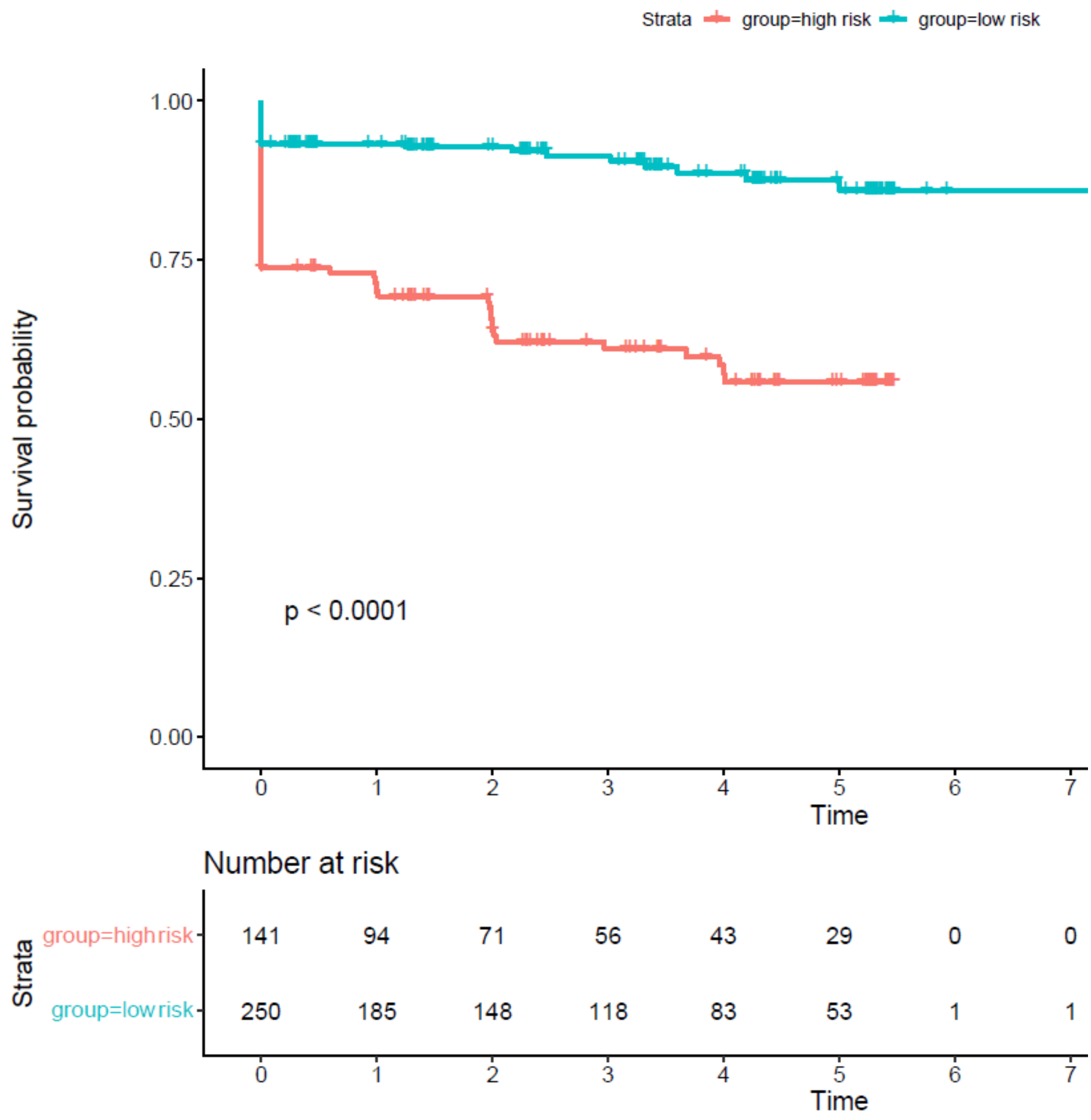
Strata
group=high risk
group=low risk
Survival probability
1.00
0.75
0.50
0.25
0.00
p < 0.0001
0
1
2
3
4
5
6
7
Time
Number at risk
Strata
group=high risk
group=low risk
141 94 71 56 43 29 0 0
250 185 148 118 83 53 1 1
Time

C. ΔArea.

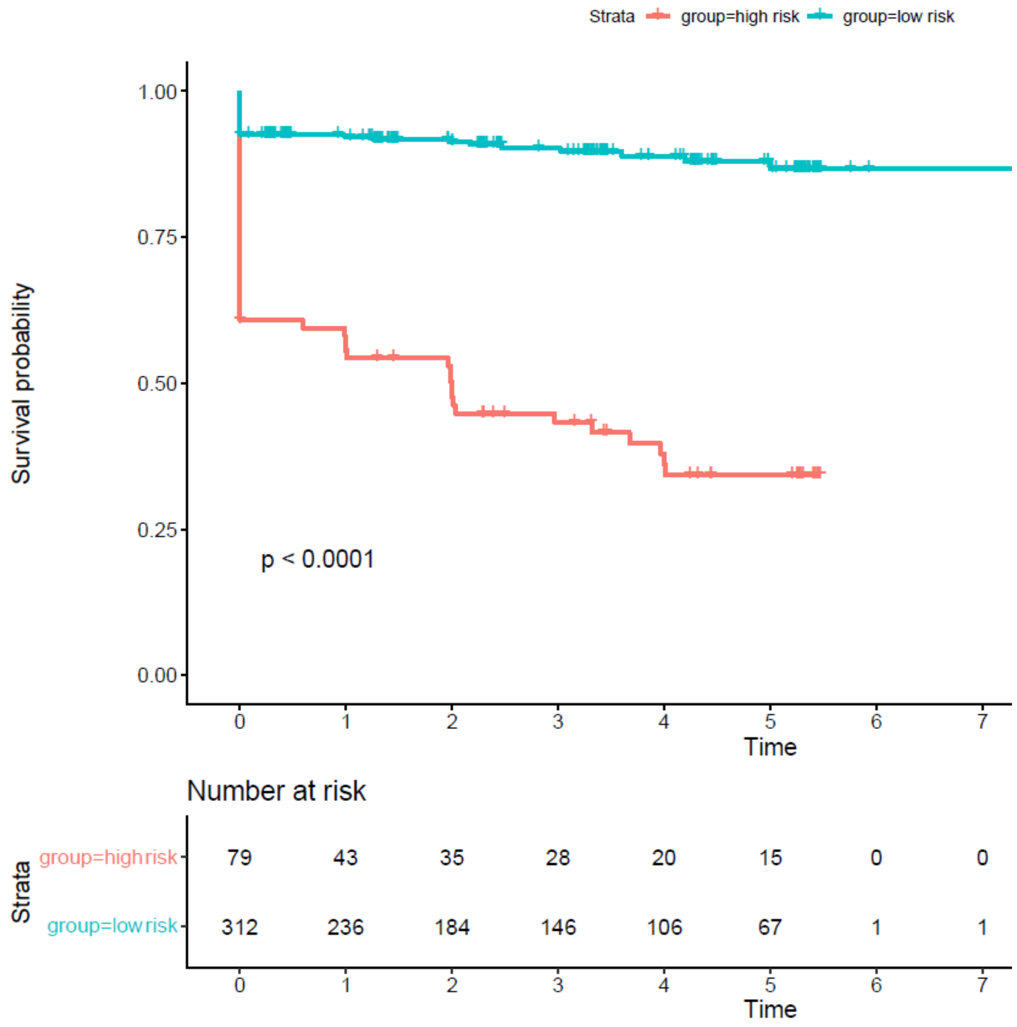

D. ΔArea*probability.
